# Dimethyl fumarate alleviates the nitroglycerin (NTG)-induced migraine in mice

**DOI:** 10.1186/s12974-020-01736-1

**Published:** 2020-02-17

**Authors:** Giovanna Casili, Marika Lanza, Alessia Filippone, Michela Campolo, Irene Paterniti, Salvatore Cuzzocrea, Emanuela Esposito

**Affiliations:** 1 Department of Chemical, Biological, Pharmaceutical and Environmental Sciences, University of Messina, Viale Ferdinando Stagno D ‘Alcontres, 31, 98166 Messina, Italy; 2 Department of Pharmacological and Physiological Science, Saint Louis University, Room M 36-1402 South Grand Blvd, St. Louis, MO 63104 USA

**Keywords:** Migraine, Dimethylfumarate, Nrf-2, NF-кb

## Abstract

**Background:**

Oxidative stress and inflammatory pathways are involved in migraine and endogenous antioxidant defense system has a role in the prevention of hyperalgesia in migraine. In this study, we aimed to evaluate the role of the most pharmacologically effective molecules among the fumaric acid esters (FAEs), dimethyl fumarate, nuclear factor E2-related factor 2/antioxidant response element (Nrf-2/ARE) pathway-mediated, in regulating the hypersensitivity in a mouse model of nitroglycerine (NTG)-induced migraine.

**Methods:**

Mice were orally administered with DMF at the doses of 10, 30, and 100 mg/kg, 5 min after NTG intraperitoneal injections. We performed histological and molecular analysis on the whole brain and behavioral tests after 4 h by NTG-migraine induction. The expression of nuclear factor kappa-light-chain-enhancer of activated B cells (NF-кB) subunit p65, nuclear factor of kappa light polypeptide gene enhancer in B-cells inhibitor alpha (IκBα), inducible nitrite oxide synthase (iNOS), cyclooxygenase 2 (COX-2), Nrf-2, manganese superoxide dismutase (Mn-SOD), and heme-oxygenase-1 (HO-1) were detected by Western blot. Tail flick, hot plate, orofacial formalin, and photophobia tests were used to evaluate migraine-like pain and migraine-related light sensitivity.

Moreover, we evaluate Nrf-2-dependent mechanism by the in vitro stimulation of cells extracted by trigeminal ganglia with diethylenetriamine/nitric oxide (DETA/NO), a nitric oxide (NO) donor. The cells were pre-treated with DMF and an antagonist of Nrf-2, trigonelline (TR) 2 h before DETA/NO stimulation.

**Results:**

DMF treatment notably reduced histological damage as showed by cresyl violet staining; also, regulating both NF-κB and Nrf-2 pathway, DMF treatment decreased the severity of inflammation and increased the protective antioxidant action. Moreover, the headache was significantly reduced. The protective effect of DMF treatment, via Nrf-2, was confirmed in in vitro studies, through inhibition of Nrf-2 by trigonelline. Cytotoxicity, iNOS, and MnSOD expression were evaluated.

**Conclusion:**

These results provided the evidence that DMF, by Nrf-2 modulation, has a protective effect on central sensitization induced by NTG, suggesting a new insight into the potential application of DMF as novel candidates in drug development for migraine.

## Introduction

Migraine is the third most common disease worldwide, with a higher prevalence in females (17.5%) than in males (8.9%) [1]. Sex differences in migraine also extend to greater symptomology, higher rate of visual auras, higher headache-related disability, and greater healthcare resource utilization by females [2]. To date, the triggering cause of migraine is unknown, but it is known that various factors, such as genetics and environmental factors, are partially involved in the debut of migraine attacks [3]. Recommended medications for the acute treatment of migraine encompass sumatriptans, nonsteroidal anti-inflammatory drugs (NSAIDs), and analgesics; particularly, the counter analgesics and non-steroidal anti-inflammatory drugs for acute mild migraine and specific prescription drugs (sumatriptans and ergot alkaloids) for acute severe migraine [4]. While it is true that sumatriptans have been the first successful mechanism-driven treatment in the field, recently, new targets involved in migraine pathogenesis have emerged and new drug classes have been studied for migraine attack therapy [5]. Recently, calcitonin gene-related peptide (CGRP) antagonists are occupying a position of redress in the treatment of migraine [6]; the discovery that the levels of the CGRP peptide increase during a migraine attack and the infusion of CGRP can provoke a migraine attack have provided evidence for the study and development of small molecule antagonists, designed to block CGRP receptor action by preventing binding of the CGRP peptide [7]. Encouraged by the efficacy of blocking CGRP for the treatment of migraine, monoclonal antibodies able to block either CGRP or its receptors were developed and tested in several preclinical modalities and the field has been very active, with now three antibodies drug approved [8, 9]. The three new FDA-approved drugs are erenumab, fremanezumab, and galcanezumab [6]. Initial programs on the development CGRP antagonists were frustrated by liver toxicity but the current outlook is very promising with five small molecule antagonists in various stages of clinical trial. Designed to decrease the number of migraine attacks, the new class of migraine drugs is metabolized differently and has fewer adverse reactions observed in clinical trials, as well as fewer warnings and precautions, compared to other approved migraine therapeutics. In general, blocking CGRP in migraine patients is seemingly both efficient and well tolerated; however, the inhibition of CGRP may pose a risk in subjects with comorbidities such as cardiovascular diseases, besides the fact that long-term effects are still unknown and that are often accompanied by high prices [10].

Migraine sufferers still have no effective and widely applicable drug treatment methods, thus the developing of more effective and safe anti-migraine agents is still a pressing task. Further studies are needed to investigate the possibility of combining different drug classes to optimize the clinical response and the potential role of the novel drugs in medication-overuse headache [5].

Though migraine causes are not understood, it demonstrated that oxidative stress is a universal migraine trigger. High levels of oxidative stress are associated with a fourfold increase in migraine risk and a recent study found that nearly all documented migraine triggers are associated with oxidative stress [11]. Also, migraine is a result of the interaction between multiple genes with environmental factors and triggers. The discovery of genes involved in this disease may lead to new insights into the molecular pathways in the pathogenesis of migraine because the discussed polymorphisms may influence the phenotypic features of migraine patients [12, 13]. Particularly, oxidative stress has been implicated in various headache disorders because it arises an imbalance between the production of reactive oxygen species (ROS) and elimination by antioxidant defense mechanisms, consequently with endogenous ROS that can cause oxidative damage to DNA, lipids, and proteins [14].

The master regulator of cellular cytoprotective responses to oxidative stress is nuclear factor erythroid-2-related factor-2 (Nrf-2) [15]. Dimethyl fumarate, DMF, the most pharmacologically effective molecule among the fumaric acid esters (FAEs) and an oral therapeutic agent for the treatment of relapsing-remitting multiple sclerosis (MS) patients [16], was discovered to impact the antioxidative stress cell machinery promoting the transcription of genes downstream to the activation of Nrf-2 [17]. In fact, it has been found that the Nrf-2/ARE pathway is the most important endogenous antioxidant defense system, and plays a critical role in regulating cellular oxidation, cell defense, and protection; moreover, increasing data points out the protective role of Nrf2/ARE pathway activation in the brain [18]. A recent study demonstrated that activation of the Nrf-2/ARE pathway inhibited the activation of trigemino vascular system (TGVS) and prevented the induction of hyperalgesia, however without specifying the underlying mechanisms of migraine [19].

Thus, the aim of this study was to investigate the role of DMF, as an activator of Nrf-2/ARE pathway, in nitroglycerin (NTG)-induced hyperalgesia and its underlying mechanisms. In fact, it has been demonstrated that NTG administration produces attacks phenotypically similar to spontaneous migraine attacks and sensitizes trigeminal and cortical structures that underlie migraine allodynia [20].

In this study, we used sumatriptan, as positive control; sumatriptan is part of triptan family that is selective agonist of serotonin 5-hydroxytryptamine serotonin (5-HT) _1B/1D_ receptors. It represents a very successful acute migraine therapy with a well-developed scientific validation, especially in reverting the behavioral hypersensitivity following NTG injection [21, 22].

Based on this finding, we speculate to consider DMF as an effective and alternative therapeutic approach to treat migraine pathology, reducing the side effects associated to traditional migraine treatments.

## Materials and methods

### In vivo study

#### Animals

CD1 mice (male 25 to 30 g, Envigo, Italy) were used for the in vivo study and Wistar rats (male 200–250 g, Envigo, Italy) were used for the in vitro study. Mice and rats were housed five per cages (five per cage) and maintained under a 12:12 h light/dark cycle at 21 ± 1 °C and 50 ± 5% humidity. Standard laboratory diet and tap water were available ad libitum. The University of Messina Review Board for the care of animals approved the study. Animal care was in compliance with Italian regulations on protection of animals used for experimental and other scientific purposes (Ministerial Decree16192) as well as with the Council Regulation (EEC) (Official Journal of the European Union L 358/112/18/1986).

#### Migraine induction and DMF administration

NTG was prepared from a stock solution of 5.0 mg/ml nitroglycerin in 30% alcohol, 30% propylene glycol, and water (American Reagent). NTG was freshly diluted in 0.9% saline to a dose of 10 mg/kg. The vehicle used in these experiments, for Sumatriptan and DMF-treated groups, was 0.9% saline. All injections were administered as a 10 mg/kg volume. Animals were treated orally with DMF at the dose of 30 mg/kg and 100 mg/kg, 5 min following NTG injection. Mice were sacrificed 4 h following NTG injection; the whole brain with rostral spinal cord was removed and then dissected in order to evaluate the following parameters: histology analysis and Western blot analysis.

In a separate set of experiments, a cohort of 20 animals from each group was observed after NTG injection, receiving DMF until the sacrifice, in order to evaluate the behavioral testing. Also, for *pre*-*treatment* groups, we used only ten animals for histological evaluation.

#### Experimental groups

Animals were divided into six groups:

1. Sham *group*: mice received saline;
2. Sham + DMF (100 mg/kg) *group*: mice received orally DMF at the dose of 100 mg/kg;
3. NTG *group*: mice received NTG (10 mg/kg) intraperitoneally;
4. NTG + sumatriptan *group*: mice received sumatriptan orally (600 μg/kg) 5 min after NTG (10 mg/kg) intraperitoneally;
5. NTG + DMF (30 mg/kg) *pre*-*treatment group*: mice received orally DMF at the dose of 30 mg/kg 2 h before NTG injection;
6. NTG + DMF (100 mg/kg) *pre*-*treatment group*: mice received orally DMF at the dose of 100 mg/kg 2 h before NTG injection;
7. NTG + DMF (30 mg/kg) *post*-*treatment group*: mice received orally DMF at the dose of 30 mg/kg 5 min after NTG injection;
8. NTG + DMF (100 mg/kg) *post*-*treatment group*: mice received orally DMF at the dose of 100 mg/kg 5 min after NTG injection.

The minimum number of mice for every technique was estimated with the statistical test “ANOVA: Fixed effect, omnibus one-way” with G-power software. This statistical test generated a sample size equal to *n* = 10 mice for each technique.

The selection of DMF treatment was based on its bioavailability, specifically the time to peak concentration of monomethylfumarate (MMF) after DMF administration is 2–2.5 h, and its half-life is approximately 1 h [23]. DMF was administered in pre- and post-treatment conditions; the doses (10, 30, and 100 mg/kg) of DMF used in this study were based on previous in in vivo experiments on metabolic and neuroinflammation diseases, dose response, and time-course studies by our laboratory. The preventive effects of DMF in migraine attacks were evaluated treating mice 2 h before NTG injections on the basis of DMF pharmacokinetics [24]. For both pre- and post-treatments, the histological effects of DMF were investigated on all three doses, but we decided to continue analyzing only DMF 30 and 100 mg/kg because DMF 10 mg/kg did not demonstrate any improvement of migraine in both pre- or post-treatment conditions. Furthermore, we showed only histological data associated to DMF alone group because toxicity or any other effects in comparison to sham control animals were not observed. The dose of sumatriptan used was based on previous experiment [22].

To better understand if the vehicle of NTG (a stock solution of 5.0 mg/ml nitroglycerin in 30% alcohol, 30% propylene glycol and water) could alter the effect of the treatments administered (Sumatriptan and DMF), in another set of experiment, mice were treated with Sumatriptan and DMF 100 mg/kg using the vehicle of NTG as vehicle, as better described in Additional file 1.

### Behavioral testing

#### Tail flick test

Warm- and cold-water tail flick tests were conducted to assess thermal allodynia. Water temperature was maintained at 46 ± 0.1 °C using a hot plate or at 15 ± 0.1 °C using crushed ice. For testing, each mice were gently wrapped in a terry cloth towel and its tail submerged 5 cm. Latency to flick or curl the tail was recorded with a 40-s cut-off [25].

#### Orofacial formalin test

The formalin test was performed according to the method of Fu et al. [26]. The mice received a subcutaneous injection of 20 μL of diluted formalin (as the formalin model group) or saline (as the control group) into the center of the right vibrissa pad. DMF (40 μl for 30 and 100 mg/kg) was injected intraperitoneally 30 min before formalin injection (as the formalin + DMF groups). After injection, the animals were immediately placed back in the test box to be observed for 45 min. Observation period was divided into 15 blocks of 3 min, and the number of seconds the animal spent in ipsilateral face rubbing or grooming was measured during phase I (0–12 min) and phase II (12–45 min) of formalin-induced pain [27]. An investigator analyzed the behavior in a blinded fashion.

#### Hot plate test

The hot plate test was carried out to assess the effects of agents on the thermal nociceptive threshold. Mice were placed on 52.58 °C hot plate. The response latency to either a hind-paw lick or jump was recorded. In the absence of a response, the animals were removed from the hot plate at 60 s to avoid tissue injury, and a 60-s latency was assigned as the response. The latency time to pain reaction was measured at 30, 60, 90, 120, and 240 min post NTG-induction [28].

#### Light/dark test

An apparatus was made to create a light/dark box used to quantify the ICHD-3 criteria of photophobia and reduced activity associated with migraine. Clear lids of the black and center gray chambers were covered with heavy black construction paper (inside ≤ 5 lx); the white chamber with clear lids served as the light portion (inside ≥ 635 lx). On test day, mice were placed into the center chamber for a 1-min acclimation period after which the guillotine-style doors were opened to allow access to the entire apparatus. Time in the light chamber and total number of photobeam breaks during a 5-min test session were recorded [29].

#### Elevated plus maze test

Anxiety is one of the common behavioral conditions in migraine, so anxiety deficits were evaluated using elevated plus maze (EPM) system, as previously described (Pellow et al. 1985). EPM consisting of two open arms and two enclosed arms, animals were placed on the centre of the maze facing an open arm and following measures scored: time spent in enclosed arms (s) and number of entrances into open arms.

#### Tissue processing and histology

Following 4 h NTG injection, animals were evaluated by an experienced histopathologist. Longitudinal sections of 7-μm thickness were processed from the brainstem at the trigeminal nucleus spinal trigeminal nucleus (SpV). Sections were then deparaffinized with xylene and then stained with cresyl violet. For cresyl violet staining, the slides were fixed as described by [30]. All sections were studied using an Axiovision Zeiss microscope (Milan, Italy). Histopathologic changes of the gray matter were scored on a five-point scale: 0, no lesion observed; 1, gray matter contained one to five eosinophilic neurons; 2, gray matter contained five to ten eosinophilics neurons; 3, gray matter contained more than ten eosinophilics neurons; 4, small infarction (less than one-third of the gray matter area); 5, large infarction (more than half of the gray matter area). The scores from all the sections of each brain were averaged to give a final score for individual mice. All the histological studies were performed in a blinded fashion [31].

#### Western blot analysis

Western blot analysis was performed on whole brain with rostral spinal cord tissues harvested 4 h after NTG injection. Cytosolic and nuclear extracts were prepared as described previously [32]. The expression of nuclear factor of kappa light polypeptide gene enhancer in B cells inhibitor alpha (IκB-α), inducible nitric oxide synthase (iNOS), cyclooxygenase 2 (COX-2), manganese superoxide dismutase (Mn-SOD), and heme-oxygenase 1 (HO-1) was quantified in cytosolic fraction. Nuclear factor kappa-light-chain-enhancer of activated B cells (NF-κB) and nuclear factor erythroid 2-related factor 2 (Nrf-2) expressions were quantified in nuclear fraction. The filters were probed with specific Abs: anti-NF-κB (1:500; Santa Cruz Biotechnology) or anti-Nrf-2 (1:500; Santa Cruz Biotechnology, SC-722) or IκB-α (1:500; Santa Cruz Biotechnology), anti-MnSOD (1:500; Millipore) or anti-HO-1 (1:500; Santa Cruz Biotechnology), anti-COX-2 (1:500; Cayman) or iNOS (1:500, Santa Cruz) in 1 × PBS, 5% w/v nonfat dried milk, 0.1% Tween-20 at 4 °C, overnight. To ascertain that blots were loaded with equal amounts of proteins, they were also incubated in the presence of the antibody against GAPDH (cytosolic fraction 1:500; Santa Cruz Biotechnology) or lamin A/C (nuclear fraction 1:500 Sigma–Aldrich Corp.) as described [31].

### In vitro study

#### Primary cell culture

Neuronal and satellite cell cultures from trigeminal ganglia were prepared from 6- to 7-day-old Wistar rats. Briefly, both trigeminal ganglia were aseptically removed as previously described [33]. Cells were mechanically dissociated using a Pasteur pipette, plated on a 25 cm^2^ flask, and incubated for a week, during which the medium was replaced twice, until reaching almost complete confluence. At this time, cells were detached from the flask by a 5-min 0.05% trypsin-EDTA (Biochrom) treatment at 37 °C, resuspended in fresh complete culture medium, and plated: in preliminary experiments to assess cell viability, 3 × 10^4^ cells were plated in a volume of 150 μl in 96-well plates. Increasing concentrations of DMF (1, 10, 30, 50, 100 μM) were used to determine the effective concentrations with minimal cytotoxicity. The DMF concentrations chosen were 1, 10, and 30 μM. In another set of experiments, 8 × 10^5^ cells were plated for 24 h, then cells were pre-treated for 2 h with 1, 10, and 30 μM DMF (based on previous 4,5- dimethylthiazol-2-yl-2,5-diphenyltetrazolium bromide colorimetric cell viability assay), following by addition of diethylenetriamine/nitric oxide (DETA/NO) (Sigma–Aldrich, St. Louis, MO), a nitric oxide (NO) donor, at the final concentration of 100 μM for 6 h [33]. The protective concentration chosen was 30 μM DMF; 1 and 10 μM DMF were not protective in DETA/NO damaged cells. In another group, cells were preincubated for 30 min with 1 μM of trigonelline (TR), a Nrf-2 inhibitor, as previously described (55) before treatment with DMF (30 μM) and the following stimulation with DETA/NO; 6 h later, cell lysates were prepared for Western blot analysis.

Cells were divided into four groups:

1. Control group (Ctr): extracted cells were cultured with normal medium;
2. Control + TR group: extracted cells were pre-treated with 1 μM TR for 30 min;
3. DETA/NO group: extracted cells were treated with 100 μM of DETA/NO;
4. DETA/NO + DMF 30 μM group: extracted cells were treated with DMF 30 μM for 2 h before addition of 100 μM of DETA/NO;
5. DETA/NO + DMF + TR group: extracted cells were pre-treated with 1 μM TR for 30 min, treated with DMF 30 μM for 2 h before addition of 100 μM of DETA/NO for 6 h.

The time and the concentration of the treatments were based on previous study [34].

#### Western blot analysis for primary cell cultures

Western blot analysis was performed as previously described [35]. The membrane was incubated overnight at 4 °C with anti-iNOS (1:500; Transduction Labs) and anti-Mn-SOD (1:500; Merck-Millipore). To ascertain that blots were loaded with equal amounts of protein lysate, they were also incubated with GAPDH antibody (1:500; Santa Cruz Biotechnology).

### Statistical analysis

All data are expressed as the mean ± SEM. Statistical analyses were performed using PRISM 5 version 5.0 (SPSS Inc., Chicago, IL, USA). Data at different time points were analyzed using a two-way analysis of variance (ANOVA) followed by Bonferroni post-hoc test. Other data were analyzed using one-way ANOVA followed by Bonferroni post-hoc test. *P* value < 0.05 was considered as statistically significant.

## Results

### Post-treatment with DMF restored the NTG-induced damage in trigeminal nucleus

**Fig. 1:**
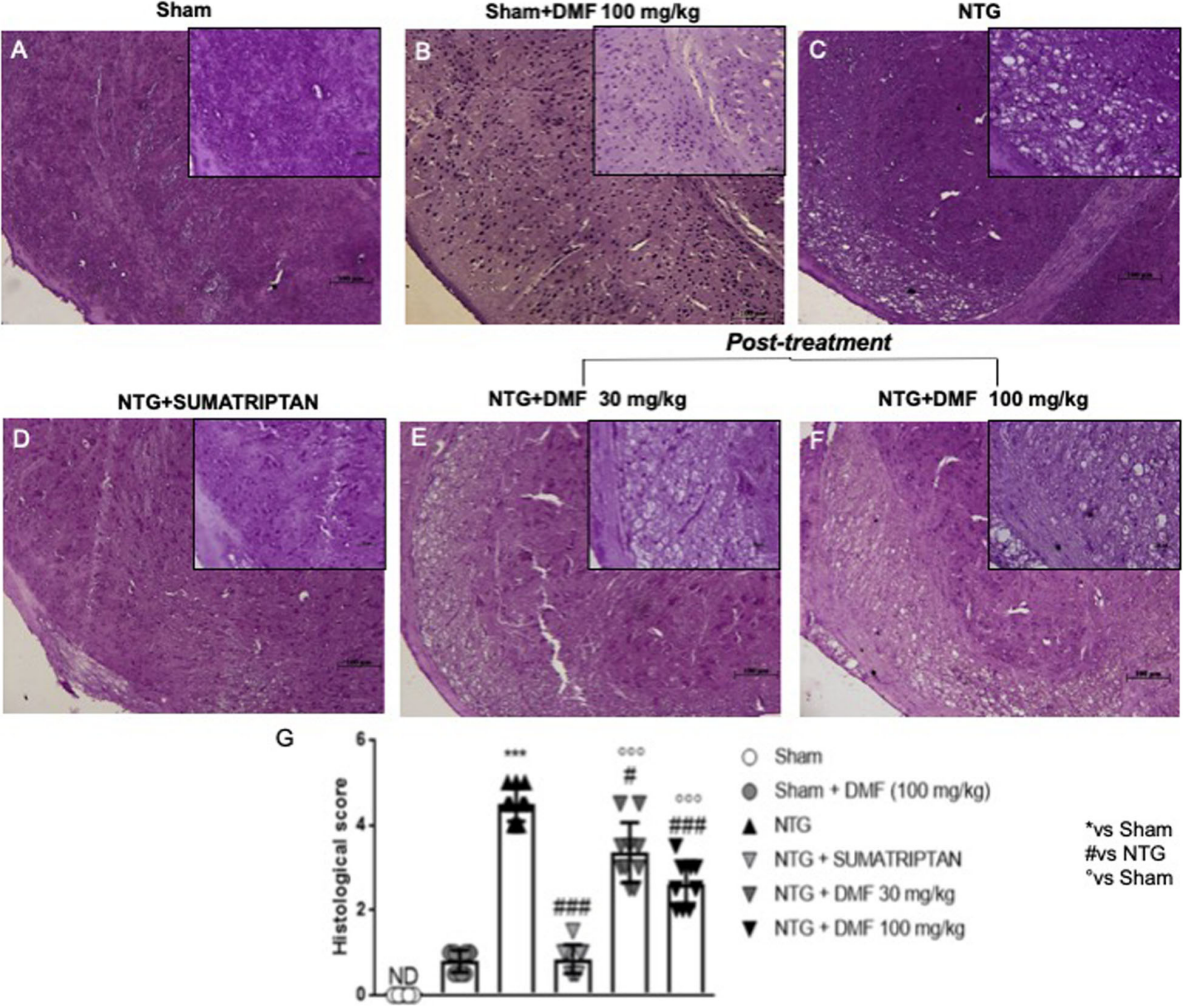
Effects of DMF treatment on NTG-induced damage. The neuronal damage in Sp5C neurons was observed in NTG-injured mice (B, B1 and F, F1) respect to control and control + sumatriptan group (respectively A, A1; C, C1; and F, F1). The treatment with DMF, mainly at the dose of 100 mg/kg, significantly restored a large number of trigeminal neurons in Sp5C area (E, E1). Data are means ± SEM of ten mice for each group. One-way ANOVA followed by Bonferroni post-test. *ND* not detectable. ***p < 0.001 vs. Sham; #*p* < 0.05 and ###*p* < 0.001vs. NTG, °°°*p* < 0.001 vs Sham. *F* value = 60.88

The symptoms that appear before the onset of migraine are related to abnormal neuronal activity in cortical and brainstem structures; particularly, it is widely accepted that trigeminal sensory information can reach the hypothalamus via multisynaptic pathways through the brainstem [36]. Central processes of sensory afferents enter the brainstem via the trigeminal tract and pass caudally while giving off collaterals that terminate mainly in lamina V of the spinal trigeminal nucleus, modulating the perception of trigeminal pain [37]. Thus, to define the NTG-induced alterations of Sp5C area, whole brain with the rostral cervical spinal cord sections were stained with cresyl violet, observing a significant neuronal damage in NTG-injured mice (Fig. 1c, see histological score Fig. 1g) respect to control and NTG + sumatriptan group (Fig. 1a, d, see histological analysis Fig. 1g). On the contrary, the post-treatment with DMF, mainly at the dose of 100 mg/kg, significantly ameliorated the cyto architecture of Sp5C area, restoring a large number of trigeminal neurons (Fig. 1f, see histological score Fig. 1g). Furthermore, mice treated with DMF 100 mg/kg (Fig. 1b, see histological score Fig. 1g), without suffering the NTG-damage, did not manifest any protective effect in comparison to sham control animals and NTG + sumatriptan group (Fig. 1a, d, see histological score Fig. 1g).

### The protective effects of DMF to reduce NTG-induced hyperalgesia

**Fig. 2:**
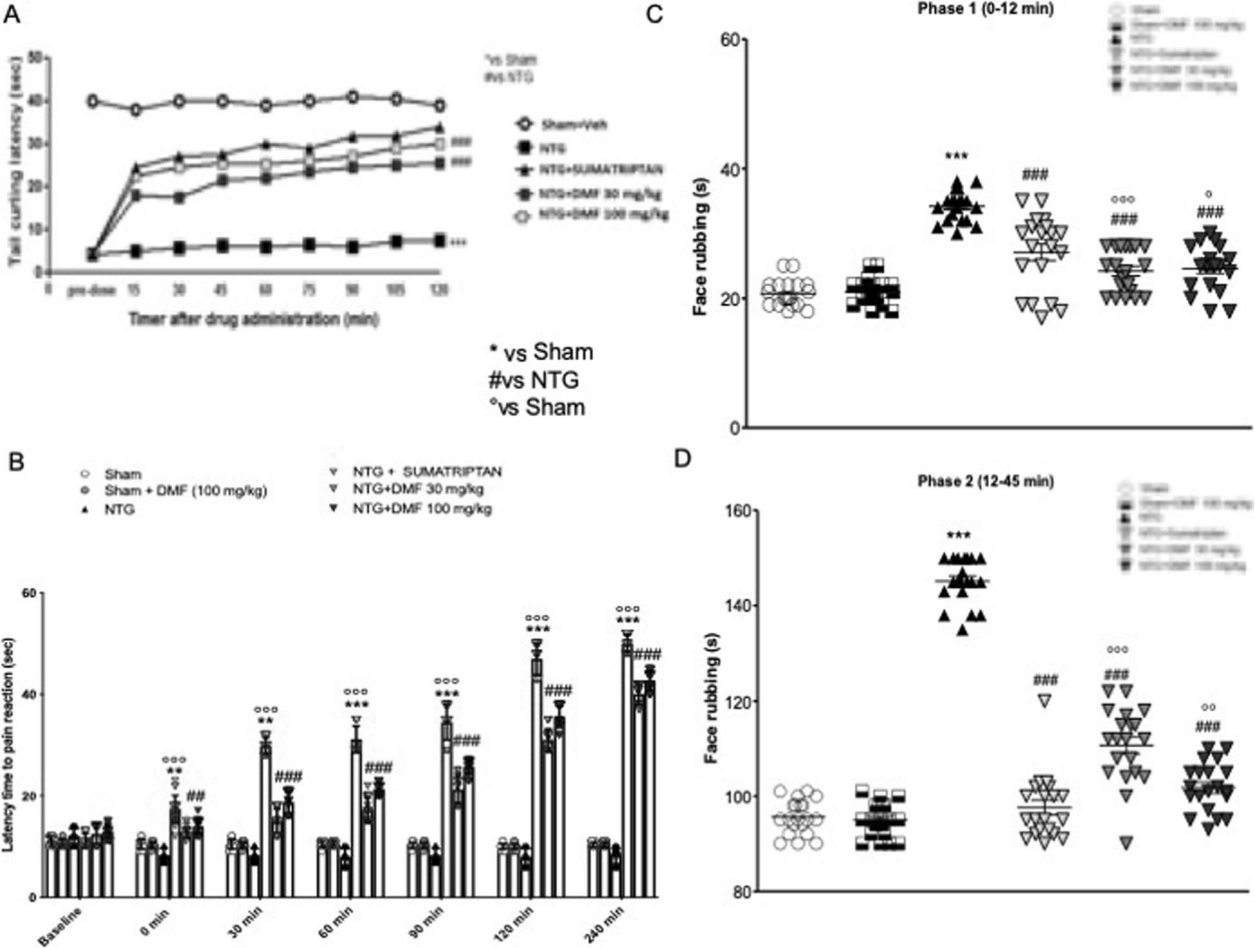
Effects of DMF treatment on NTG-induced hyperalgesia. In tail flick test, DMF treatment at 30 and 100 mg/kg administered after NTG vehicle, caused a significant increase in tail curling latency (**a**) respect to NTG group (**a**); F values for treatment = 77.24. *F* value for time = 6.749. In Hot plate test, i.p. injection of NTG increased thermal hypersensitivity in a time-dependent manner (**b**), while DMF treatment, at both doses of 30 and 100 mg/kg, significantly increased the latency time to pain reaction related to the increase in time up to 240 min after NTG injection (**b**); *F* value = 8.468. In formalin test, NTG administration significantly increased the total number of flinches/shakes in phase I and II respect to control groups (**c**, **d**), while DMF treatment at 30 and 100 mg/kg significantly alleviated the nociceptive behavior induced by NTG administration during phase I and II of the test (**c**, **d**); *F* value (**c**) = 241.4; *F* value (**d**) = 148.3. Data are means ± SEM of 20 mice for each group. One-way ANOVA and two-way ANOVA followed by Bonferroni post-test. ***p < 0.001 and **p < 0.01 vs. Sham; ##*p* < 0.01 and ###*p* < 0.001vs. NTG. °°°*p* < 0.001, °°*p* < 0.01, and °*p* < 0.001 vs. Sham

NTG-evoked hyperalgesia in mice has been developed as a model for sensory hypersensitivity associated with migraine [38]. The tail flick test is a thermal hyperalgesia test in which the tail of the animal is subjected to a heat source, removing spontaneously the tail (“tail flick”) when the fell situation become uncomfortable. In this study, DMF treatment administered after NTG caused a significant increase in tail flick latency, suggesting a DMF-mediated antinociceptive effect (Fig. 2a). Moreover, injection of NTG elicits thermal hypersensitivity in a time-dependent manner; thus, in this study, we evaluated the effect of DMF and Sumatriptan treatments on NTG-induced thermal hypersensitivity using the Hot plate test (Fig. 2b). The result highlighted that DMF treatment, at both doses of 30 and 100 mg/kg, significantly increased the latency time to pain reaction related to the increase in time from 0 (starting time of NTG injection) up to 240 min; furthermore, Sumatriptan treatment, as negative control, has increased even more the latency time to pain (Fig. 2b). In sham control and in sham + DMF-treated mice, the latency time to pain remained constant over time compared to a decrement observed in NTG-damaged mice starting from time 0 (Fig. 2b). Indeed, the pathophysiological mechanisms involved with migraine are suggestive of an increased and prolonged hyperexcitability to stimuli, especially within the trigeminal distribution. In the orofacial formalin test, total time spent in the face rubbing evoked by formalin injection was counted in phases 1 (Fig. 2c) and 2 (Fig. 2d) of the test. NTG administration significantly increased the total time of rubbing in phase II of formalin test, while DMF administration, at both doses of 30 and 100 mg/kg, significantly reduced the nociceptive score (face-rubbing time) in both phases of the orofacial formalin test (Fig. 2c, d).

### The role of DMF in the comorbidity between migraine and anxiety

**Fig. 3:**
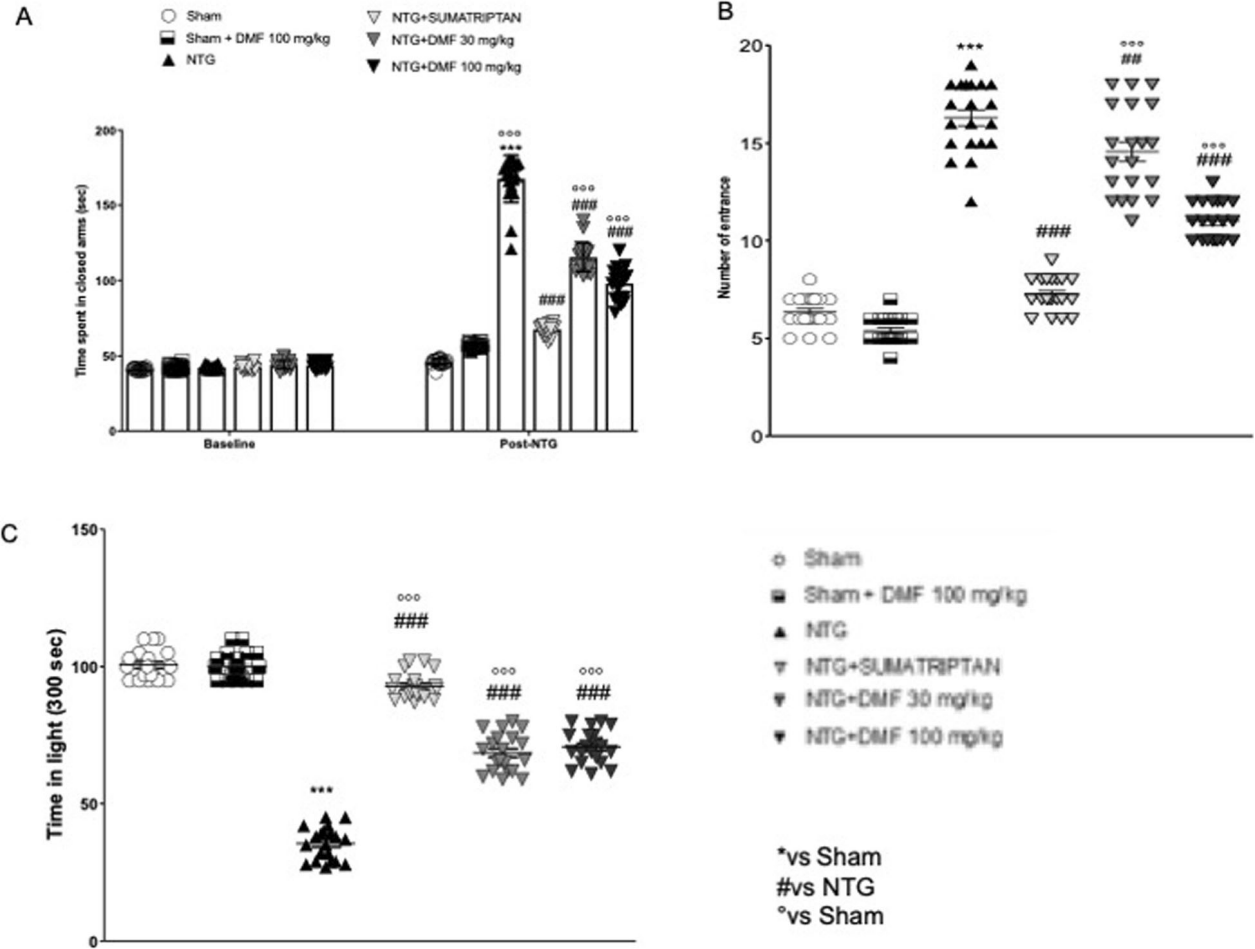
Effects of DMF treatment on NTG-induced anxiety and photophobia. EPM test was used to evaluate anxiety. NTG-injected mice showed anxiety stopping in closed arm (**a**), while DMF-treated mice spent more time in open arm (**a**); *F* values for treatment F (5, 798) = 4322; for time, *F* (6, 798) = 1360. In the same way, in NTG-injected mice, the number of entries in closed arm increased (**b**), while DMF treatment significantly augmented the residence time in open arm (**b**). In photophobia test, displaying migraine-like behaviors showed a strong tendency to stay longer in the dark zone respect to control mice (**c**), while NTG-injected mice, treated with DMF stayed more in the light zone (**c**). Data are means ± SEM of 20 mice for each group. One-way ANOVA and two-way ANOVA followed by Bonferroni post-test. *ND* not detectable. ***p < 0.001 vs. Sham; ##*p* < 0.01 and ###*p* < 0.001 vs. NTG. °°°*p* < 0.001 vs. Sham

Anxiety and mood disorders have been shown to be the most relevant psychiatric comorbidities associated with migraine, influencing disease prevalence and clinical outcomes [39]. Studies reported that mood and anxiety disorders are two to ten times more common in migraineurs than in the general population [40]. In this study, the time in closed arm and number of closed arm entries on the EPM test were used to evaluate anxiety. NTG-injected mice showed anxiety and fear to explore and they stationed in closed arm (Fig. 3a), while DMF-treated mice were encouraged and started to spend more time in open arm (Fig. 3a). In the same way, in migraine-affected mice, the number of entries in closed arm significantly increased (Fig. 3b) respect to control and sumatriptan groups (Fig. 3b); DMF treatment, mainly at 100 mg/kg, reduced the number of entries in closed arm and augmented the permanence time in open arm (Fig. 3b). Indeed, a common and debilitating symptom often present in headache disorders is photophobia, related to pain caused by the trigeminal system alteration [41]. In this study, mice displaying migraine-like behaviors showed a strong tendency to stay longer in the dark zone respect to control mice and sumatriptan-treated mice (Fig. 3c). Conversely, NTG-injected mice, treated with DMF at both doses of 30 and 100 mg/kg, started to leave the dark area and stayed more in the light zone (Fig. 3c).

### The role of DMF on antioxidant system in NTG-induced migraine

**Fig. 4:**
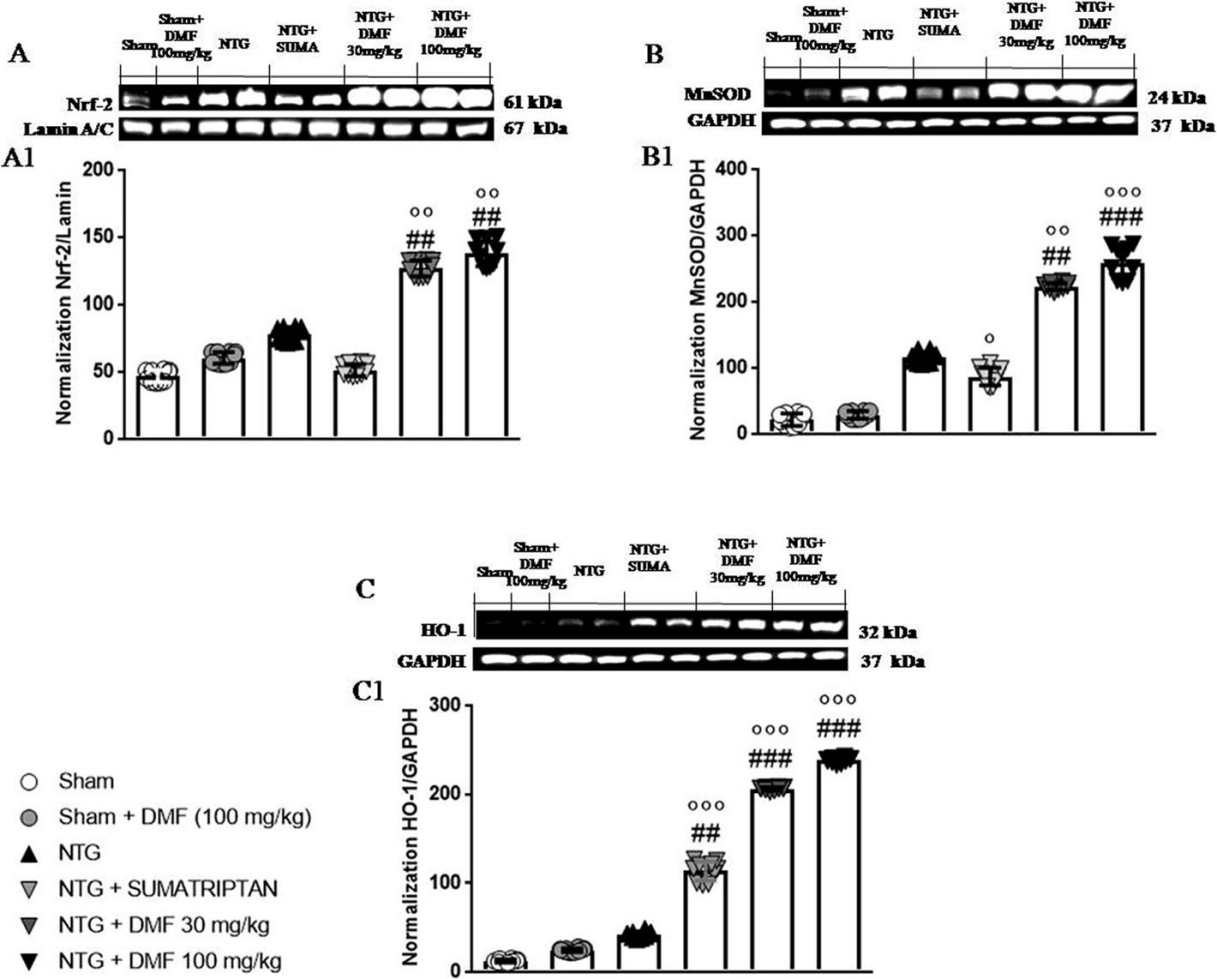
Effects of DMF on antioxidant system in NTG-induced migraine. The expression of Nrf-2, Mn-SOD, and HO-1was observed by Western blot analysis in whole brain with the rostral cervical spinal cord samples of mice, 4 h after NTG-injections. Respectively, nuclear Nrf-2 expression increased following NTG-injection as compared to sham and sumatriptan groups (A), while DMF administration, at 30 and 100 mg/kg, upregulated Nrf-2 activity (A); *F* value = 438.9. The same result was obtained for Mn-SOD expression (B); *F* value = 11.69. The levels of HO-1 were notably increased following NTG-damage compared to control groups (C), while DMF treatment, at 30 and 100 mg/kg, significantly up-regulated Mn-SOD and HO-1 expression (B and C); *F* value = 7.79. Data are means ± SEM of ten mice for each group. A representative blot of lysates obtained from each group is shown and densitometry analysis of all animals is reported (*n* = 10 mice from each group). One-way ANOVA followed by Bonferroni post-test. ##*p* < 0.01 and ###*p* < 0.01 vs. NTG; °*p* < 0.05, °°*p* < 0.01 and °°°*p* < 0.001 vs. Sham

Oxidative stress is also believed to play a role in the pathogenesis of migraine [42]. Nrf-2 is a transcription factor with strong antioxidant effects, which protects neurons from ROS-induced damage. We evaluated the effects of DMF on Nrf-2 pathway and related proteins, in whole brain with the rostral cervical spinal cord samples, by Western blot analysis. Respectively, nuclear Nrf-2 expression showed a tendency to increase following NTG-injection as compared to sham, sham + DMF and sumatriptan groups (Fig. 4(A), see densitometric analysis Fig. 4(A1)); DMF administration, at both doses of 30 and 100 mg/kg, after migraine induction, up-regulated the activities of Nrf-2 (Fig. 4(A), see densitometric analysis Fig. 4(A1)). Moreover, DMF treatment, at both doses of 30 and 100 mg/kg, after NTG injections, upregulated the activity of Mn-SOD (Fig. 4(B), see densitometric analysis Fig. 4(B1)), respect the basal expression in controls groups (Fig. 4(B), see densitometric analysis Fig. 4(B1)). Indeed, the levels of HO-1 were increased following NTG-damage compared to control and sumatriptan groups (Fig. 4(C), see densitometric analysis Fig. 4(C1)). Interestingly, treatment with DMF 30 and 100 mg/kg significantly upregulated, in the same way at both doses, HO-1 expression (Fig. 4(C), see densitometric analysis Fig. 4(C1)). Furthermore, to assess whether the NTG-vehicle (30% alcohol, 30% propylene glycol, and water) could influence the antioxidant effects of DMF or Sumatriptan on control group, the expression of Nrf-2, Mn-SOD, and HO-1 was evaluate by Western blot analysis in whole brain with the rostral cervical spinal cord samples from NTG-vehicle-treated mice, as described in more details (see Additional file 1 and Additional file 2: Figure S2).

### The effects of DMF on NF-κB inflammatory pathway in NTG-induced migraine

**Fig. 5:**
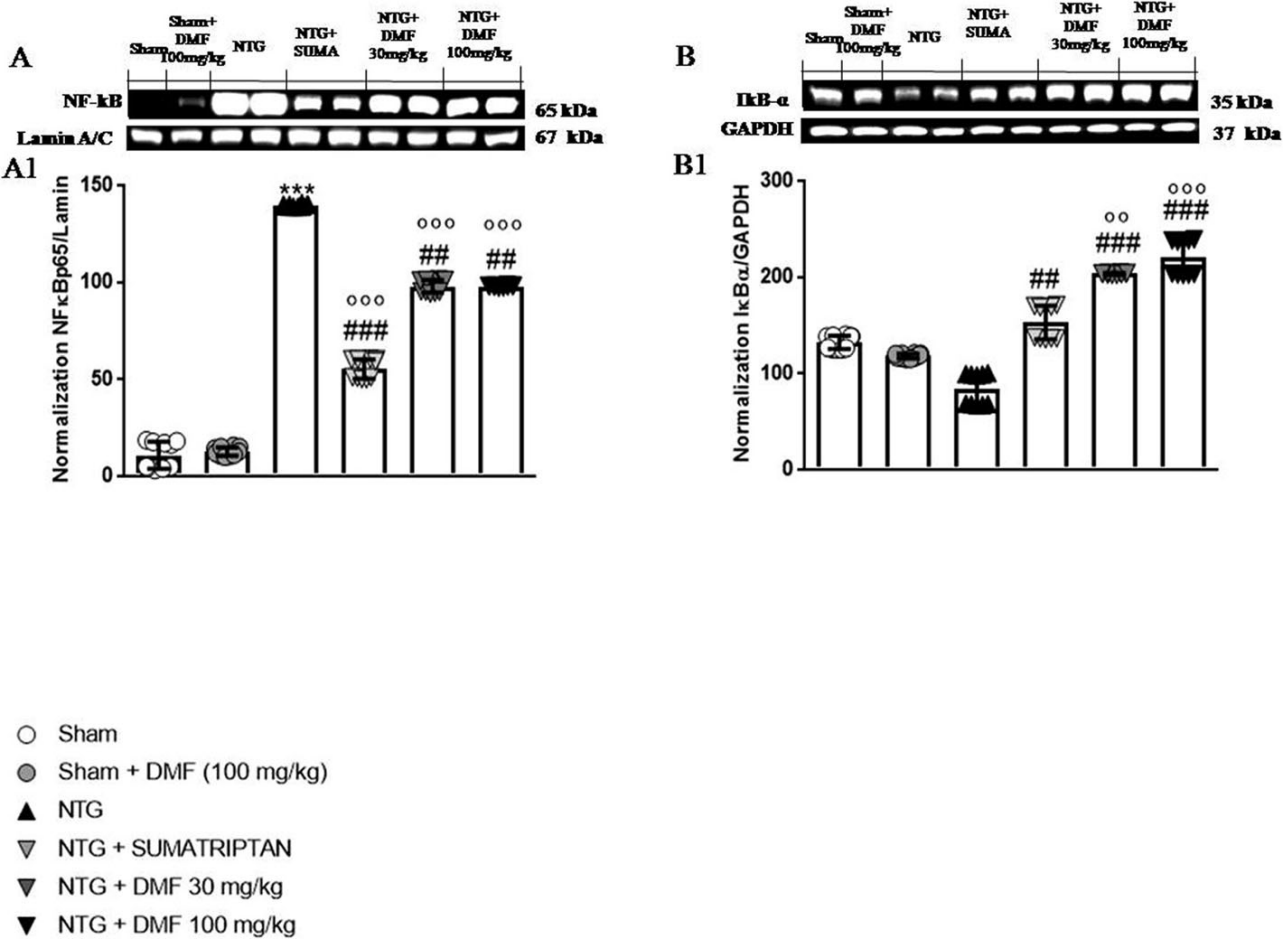
Effects of DMF on NF-κB inflammatory pathway in NTG-induced migraine. The expression of NF-κB and IκB-α was observed by Western blot analysis, in whole brain with the rostral cervical spinal cord samples of mice, 4 h after NTG-injections. We observed that NTG-injections caused a significant NF-κB nuclear translocation, almost completely inhibited by DMF 30 and 100 mg/kg treatment (A); *F* value = 165.6. Also, DMF treatment, only at the dose of 100 mg/kg, significantly reduced IκB-α cytosolic phosphorylation (B), while in NTG-injected mice the levels of IκB-α cytosolic phosphorylated were significantly increased respect to control groups (B); *F* value = 166.2. Data are means ± SEM of ten mice for each group. A representative blot of lysates obtained from each group is shown and densitometry analysis of all animals is reported (*n* = 10 mice from each group). One-way ANOVA followed by Bonferroni post-test. ***p < 0.001 vs. Sham; ##*p* < 0.01 and ###*p* < 0.001 vs. NTG. One-way ANOVA followed by Bonferroni post-test. ##*p* < 0.01 and ###*p* < 0.01 vs. NTG; °*p* < 0.05, °°*p* < 0.01, and °°°*p* < 0.001 vs. Sham

**Fig. 6:**
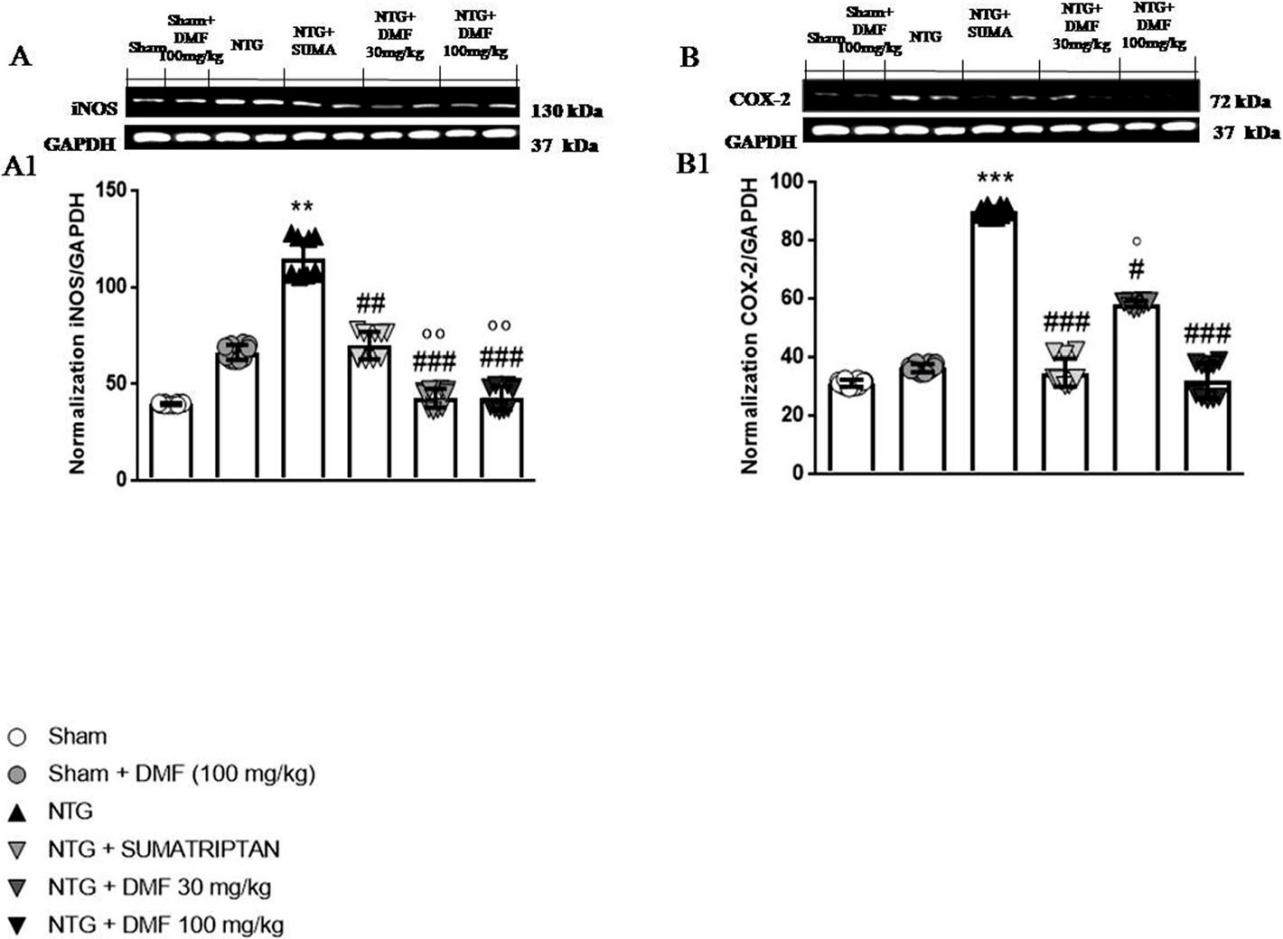
Effects of DMF on iNOS and COX-2 expression in NTG-induced migraine. The expression of iNOS and COX-2 was observed by Western blot analysis, in whole brain with the rostral cervical spinal cord samples of mice, 4 h after NTG-injections. A significant increase in iNOS expression was observed in NTG group (A), while DMF treatment significantly reduced iNOS expression at 30 and 100 mg/kg (A); *F* value = 18.92. Alike, COX-2 expression was significantly elevated in NTG-injected mice in comparison to controls (B). The rise in COX-2 expression induced by NTG was considerably blocked by treatment with DMF 30 and 100 mg/kg (B); *F* value = 37.47. Data are means ± SEM of ten mice for each group. A representative blot of lysates obtained from each group is shown and densitometry analysis of all animals is reported (*n* = 10 mice from each group). One-way ANOVA followed by Bonferroni post-test. **p < 0.01 and ***p < 0.001 vs. Sham; #*p* < 0.05, ##*p* < 0.01, and ###*p* < 0.001 vs. NTG; °*p* < 0.05 and °°*p* < 0.01 vs. Sham

Oxidative stress-induced acute inflammatory responses may play an important role in the pathogenesis of acute pain arising from migraine [14]. To evaluate the anti-neuro inflammatory effect by which DMF treatment may regulate cytokines expression during headache attack, we assessed the expression of NF-κB, IκB-α, iNOS, and COX-2 in whole brain with the rostral cervical spinal cord samples by Western blot analysis. We observed that NTG-injections caused a significant NF-κB nuclear translocation, almost completely inhibited by DMF 30 and 100 mg/kg treatments, as observed in Fig. 5(A and A1). Also, DMF treatment, only at the dose of 100 mg/kg, significantly reduced IκB-α cytosolic degradation respect to NTG-injected mice (Fig. 5(B), see densitometric analysis Fig. 5(B1)); still, the treatment with sumatriptan significantly reduced the cytosolic degradation of IκB-α (Fig. 5(B), see densitometric analysis Fig. 5(B1)). In sham and sham + DMF groups, the levels of IκB-α were significantly high respect to NTG group (Fig. 5(B), see densitometric analysis (Fig. 5(B1)). Indeed, a significant increase in iNOS expression was observed in the whole brain with the rostral cervical spinal cord samples from mice injected with NTG (Fig. 6(A), see densitometric analysis Fig. 6(A1)), while DMF treatment significantly reduced iNOS expression at 30 and 100 mg/kg (Fig. 6(A), see densitometric analysis Fig. 6(A1)) such as sumatriptan administration (Fig. 6(A), see densitometric analysis Fig. 6(A1)). COX-2 expression was significantly elevated in NTG-injected mice in comparison to control and sumatriptan groups (Fig. 6(B), see densitometric analysis Fig. 6(B1)). The rise in COX-2 expression induced by NTG was considerably blocked by treatment with DMF 30 and 100 mg/kg (Fig. 6(B), see densitometric analysis Fig. 6(B1)). Furthermore, to assess whether the NTG-vehicle (30% alcohol, 30% propylene glycol, and water) could influence the anti-inflammatory effects of DMF or Sumatriptan on control group, the expression of NF-κB, IκB-α, iNOS, and COX-2 was performed by Western blot analysis in whole brain with the rostral cervical spinal cord samples from NTG-vehicle-treated mice, as described in more details (see Additional file 1 and Additional file 2: Figure S2).

### Evaluation of Nrf-2-dependent mechanism of DMF in vitro

**Fig. 7:**
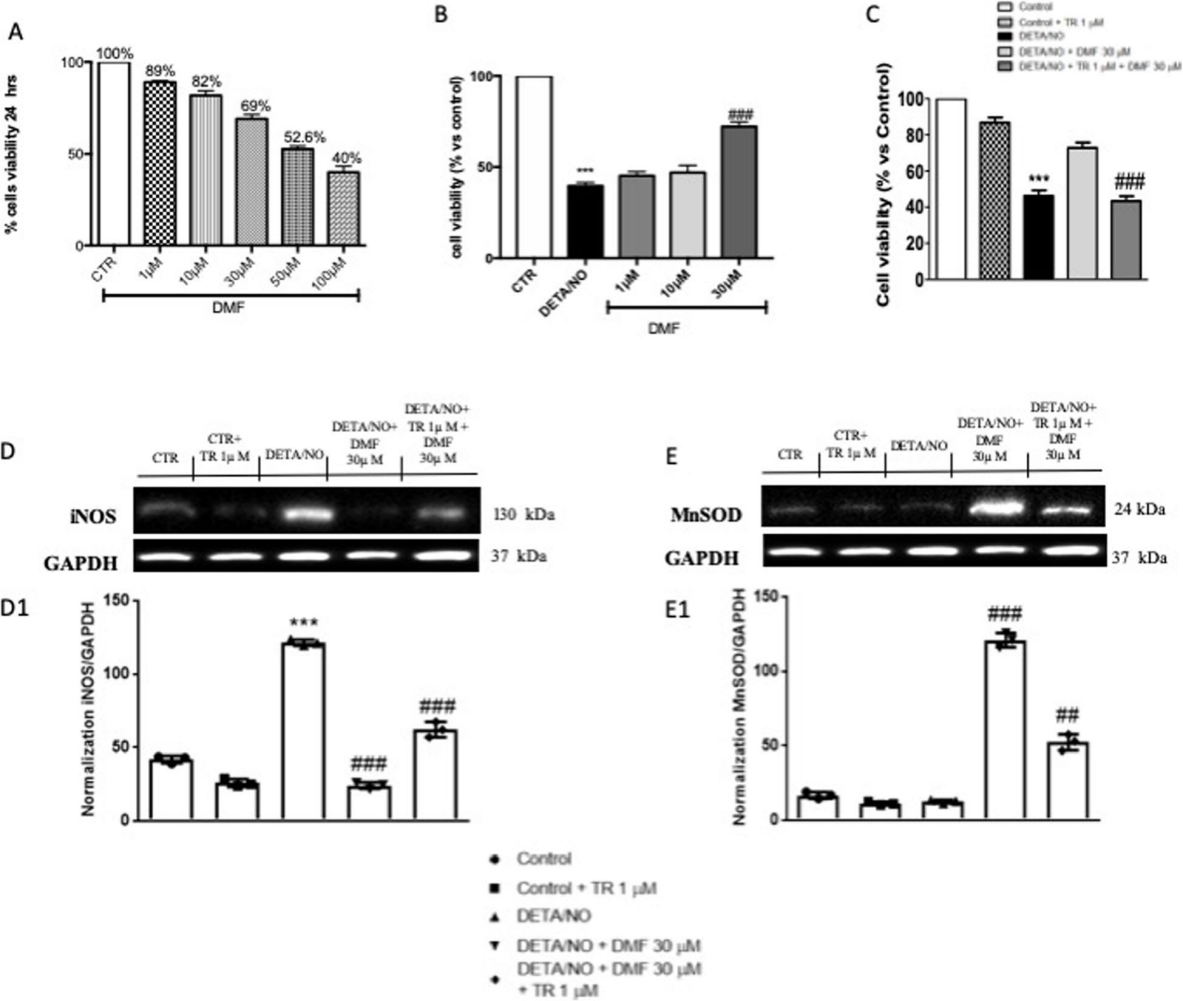
Nrf-2 dependent mechanism of DMF. Cell death was assessed 24 h after treatment with increased concentrations of DMF (1–10–30–50–100 μM); the concentration of DMF at 1–10–30 μM resulted not cytotoxic (A). DETA/NO 100 μM significantly reduced cell viability compared with the control (Ctr); pre-treatment with DMF at 30 μM significantly prevented cell death (B). Also, co-incubation of DMF 30 μM with TR (1 μM), antagonized DMF cytoprotective effect (C). Western blot analysis demonstrated a significantly increasing of iNOS expression in DETA/NO stimulated cells. However, the pre-treatment with 30 μM DMF decreased iNOS expression (D), that was abolished in the presence of 1 μM TR (D). In the same way, the decreased Mn-SOD expression seen in DETA/NO-treated cells was recovered following pre-treatment with 30 μM DMF (E). TR inhibited DMF protective action (E). Data and figures are representative of at least three independent experiments. (B) ***p < 0.001 versus Ctr; ###*p* < 0.001 versus DETA/NO (C) ***p < 0.001 versus Ctr; ###*p* < 0.001 versus DETA/NO; °°°*p* < 0.001 versus Ctr; (D1) ***p < 0.001 versus Ctr; ###*p* < 0.001 versus DETA/NO; °°°*p* < 0.001 versus Ctr; (E1) ##*p* < 0.01 and ###*p* < 0.001 versus DETA/NO. *F* value (A) = 111.8; *F* value (B) = 101.2; *F* value (C) = 94.53; *F* value (D) = 166; *F* value (E) = 185.3

The preliminary step was to evaluate DMF effect on cell viability; neurons and satellite cell extracted from trigeminal ganglia were incubated with increasing concentrations of DMF (1–10–30–50–100 μM). Cell viability assessed after 24 h showed that only DMF concentrations of 1, 10, and 30 μM lacked cytotoxicity (Fig. 7(A)). The stimulation of cells with 100 μM of DETA/NO significantly reduced viability, whereas pre-treatment with 30 μM DMF, 2 h before DETA/NO, significantly reduced cell death compared with the DETA/NO group and the other concentrations (Fig. 7(B)), thus demonstrating that DMF at 30 μM represented the most effective concentration. To confirm Nrf-2-dependent mechanism of DMF, cells from trigeminal ganglia were stimulated with the Nrf-2 antagonist TR (1 μM), 30 min before DMF treatment and the stimulation with DETA/NO. TR notably inhibited the DMF cytoprotective effect compared with the DETA/NO when cells are pre-treated before damage (Fig. 7(C)), while in cells treated only with TR, without DETA/NO damage, cell viability was similar to control (Fig. 7(C)). Moreover, to confirm the Nrf-2-mediated mechanism, to counteract inflammation and oxidative stress, we evaluated the expression of iNOS and Mn-SOD in trigeminal extracted cells. Western blot analysis demonstrated that iNOS expression was significantly increased in DETA/NO group compared with the control and TR alone group, while the pre-treatment with 30 μM DMF decreased iNOS expression (Fig. 7(D), see densitometric analysis D1). However, TR abolished DMF protective effect against DETA/NO. In the same way, the incubation with 1 μM of TR increased cells susceptibility to DETA/NO damage, lowering Mn-SOD expression (Fig. 7(E), see densitometric analysis E1). Instead, cells treated with TR, without DETA/NO damage, expressed MnSOD levels similar to control group (Fig. 7(E), see densitometric analysis E1).

### The effects of pre-treatment with DMF in NTG-induced damage

**Fig. 8:**
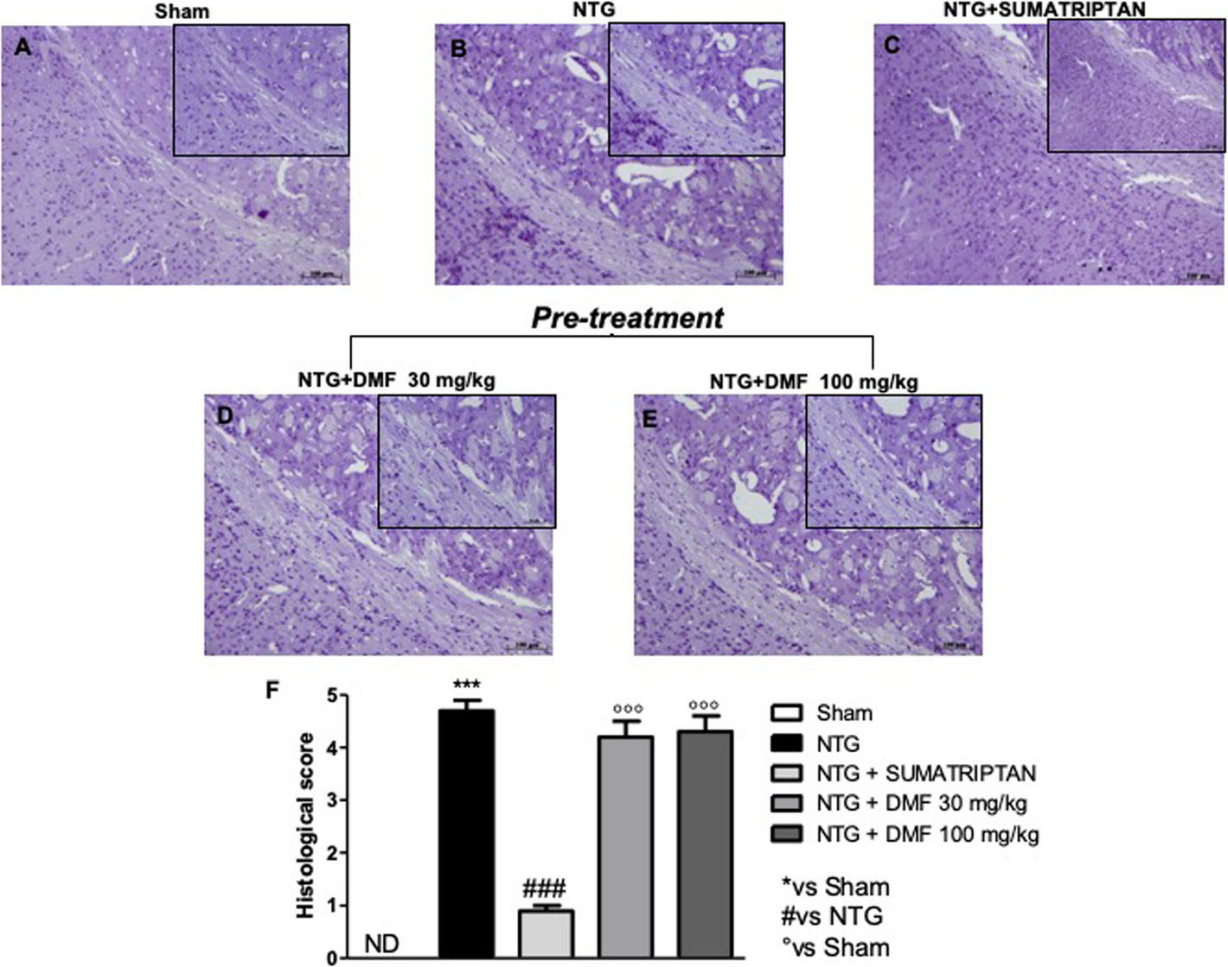
Effects of DMF pre-treatment on NTG-induced damage. The neuronal damage in Sp5C neurons was observed in NTG-injured mice (B, B1 and F, F1) respect to control (A, A1 and F, F1). The pre-treatment with DMF did not restored damage in trigeminal Sp5C area (D, D1; E, E1 and F, F1). Data are means ± SEM of ten mice for each group. One-way ANOVA followed by Bonferroni post-test. *ND* not detectable. ***p < 0.001 vs. Sham; ###*p* < 0.001vs. NTG, °°°*p* < 0.001 vs. Sham. F value = 104.7

To better understand if DMF treatment could prevent damage caused by NTG, mice were pre-treated with DMF 2 h before NTG injections (Fig. 8). Pre-treatment with DMF, at both doses of 30 and 100 mg/kg, has not showed some preventive effects from the damage induced by NTG (Fig. 8d, e, see histological score Fig. 8f).

## Discussion

Migraine represents one of the most disabling disorders [43]; therefore, medical approach should be individualized for adequate treatment of the migraine and other vascular risk factors. Several studies have shown that oxidative stress plays a role in central sensitization [44]. There are many factors that lead to the generation of oxidants in migraine; these could be explained by the metabolic changes in the cerebral cortex associated with intracellular calcium overload during cortical spreading depression (CSD) that bring to an increasing oxygen necessity which could induce oxidative stress [45, 46]. Local changes of cerebral blood flow may enhance the generation and release of free radicals, thus causing increased accumulation of lipid peroxidation by-products in blood [47, 48]. However, there are some conflicting data on the significance of oxidative stress in migraine [49, 50]. The activity of antioxidant defense system is difficult to explain; therefore, a depth study may provide the desired information on the role of oxidative stress in migraine.

It has been found that Nrf2/ARE pathway is the most important endogenous antioxidant defense system and plays a critical role in regulating cellular oxidation, cell defense and protection [51]; moreover, Nrf-2 signaling cascade modulates both inflammation and oxidative stress. There are many drugs, already used clinically, that activate Nrf-2 system, including fumaric acid ester DMF [52]. DMF is an orally administered fumarate ester, approved as first-line monotherapy in early stage of MS [53]. Because DMF is rapidly and completely hydrolyzed by esterase, before reaching the systemic circulation, the pharmacologic activity of DMF is due to its active metabolite, monomethyl fumarate (MMF). Although nowadays the precise mechanism of action of DMF is not completely characterized, is thought to exert neuroprotective effects by activating Nrf-2 transcriptional pathway [18]. DMF was discovered to impact the antioxidative stress cell machinery promoting the transcription of genes downstream to the activation of Nrf-2, which besides immune regulatory effects [17].

Recently, studies demonstrated that DMF is highly active to counteract oxidative stress and inflammation by activating the Nrf-2 genetic program to promote axonal regeneration and neurological recovery in MS as well as in other neurodegenerative diseases [54], demonstrating that DMF can alleviate early brain injury and secondary cognitive deficits in experimental subarachnoid hemorrhage [55].

Moreover, the role of Nrf-2 pathway was recently observed in in vivo model of headache demonstrating that the modulation of the Nrf2/ARE pathway inhibited the activation of trigeminus vascular system (TGVS) and prevented the induction of hyperalgesia [19]. In support of this finding, we investigated the neuroprotective effect of DMF in NTG-induced hyperalgesia through the modulation of Nrf2/ARE pathway and its underlying mechanism.

The effect of DMF on analgesia was observed by the hot plate test, tail flick, and orofacial formalin test, which are standard behavioral models for the assessment of analgesia [56]. Our result showed the analgesic role of DMF thought a significant reduction of mechanical allodynia caused by NTG administrations, relieving stimulus-evoked spontaneous nociception. Moreover, in acute and chronic hyperalgesia conditions, DMF was able to counteract peripherally pain, as evaluated by the reduction of total time of rubbing in orofacial formalin test.

The primary headache, particularly migraine, has a bidirectional relationship with depression and anxiety. Patients with depression have a more than threefold relative risk of developing migraine compared with non-depressed patients. Similarly, migraineurs have a more than threefold relative risk of developing depression compared with patients without migraine [57]. The EPM test, used in this study, quantified the anxiety-like behavior associated with repeated migraine attacks demonstrating the importance of DMF treatment to reduce the rates of anxiety and depression.

Furthermore, the perception of migraine headache is intensified during exposure to light [58]; in fact, the migraine photophobia is experienced by nearly 90% of migraineurs with normal eyesight, and depends on photic signals from the eye that converge on trigemino vascular neurons somewhere along its path. In this study, we showed that NTG injection caused light aversion in mice that was attenuated by DMF treatment, in a dose-dependent manner.

The most common migraine-associated symptoms (nausea, throbbing pain, photophobia) originate from activation of nociceptors innervating pial, arachnoid and dural blood vessels. Central processes of meningeal sensory afferents enter the brainstem via the trigeminal tract and pass caudally while giving off collaterals that terminate in the spinal trigeminal nucleus (Sp5C) and upper cervical spinal cord. Anatomical and electrophysiological studies have shown that the nociceptive innervation consists of unmyelinated (C-fibers) and thinly myelinated (Aδ fibers) axons containing vasoactive neuropeptides such as substance P (SP) and calcitonin gene-related peptide (CGRP), that originate in the trigeminal ganglion [59]. Preclinical data suggested that primary headaches are initiated by the activation of meningeal perivascular nociceptors that in turn activate brainstem trigeminovascular (Sp5C) neurons [60]. A large number of endogenous inflammatory mediators, believed to be released during migraine, are capable of activating and sensitizing peripheral and central Sp5C neurons, particularly the sensitization of second-order neurons in the Sp5C mediates cephalic allodynia as well as muscle tenderness [61]. These meningeal nociceptors converge on trigemino vascular Sp5C neurons that receive additional input from the adjacent skin and muscles [62, 63]. In this study, we observed that DMF significantly reduced the Sp5C degeneration induced by NTG-administration, resulting in a reduction in referred pain perception caused by the resultant convergence of intracranial (visceral) and extracranial (somatic) primary afferents onto Sp5C neurons.

Central sensitization associated with activation of NF-κB in the trigeminal cervical complex (TNC) is reported to be involved in the pathogenesis of migraine [19]. NF-κB is believed to be implicated in multiple signaling pathways in variable headache settings and, particularly, Li et al. showed that the impairments due to NTG-induced migraine are caused by the activation of NF-κB signal transduction pathway [64]. Also, the activation of NF-κB transcriptional activity in brain nuclei that are relevant for pain transmission observed in an animal model of migraine suggested a potential new avenue for the development of anti-migraine drugs [65]. In the past, it was demonstrated that targeting the inflammatory response by selectively inhibiting NF-κB offered a promising therapeutic approach for the treatment of headache [66]. Recently, in a study on the upregulation of inflammatory gene transcripts in chronic migraineurs, it has been discovered that three of the four genes that are abnormally expressed in chronic migraine patients—the NF-κBIA, the TNFAIP3, and the ILR2—are tightly related to the NF-κB family pathway, suggesting that suppression of NF-κB activation is critical in resolving the inflammation and consequently in reducing the pain involved in the pathophysiology of the studied chronic migraine patients [67]. Collectively, the findings point to a dual mechanism in headache: the first is activation of proinflammatory pathways and the second is suppression of pathways involved in resolving the inflammation. Particularly, in headache, neurogenic inflammation is responsible of the characteristic symptoms as pain and sensitization, because mast cells that reside close to primary nociceptive neurons are capable of triggering local inflammation [68].

Recently, it has been demonstrated that DMF induced Nrf-2 expression suppresses the NF-κB mediated inflammatory pathway [69]. Moreover, the identification of NF-κB binding cites in the promoter region of the Nrf-2 gene suggests cross talk between these two regulators of inflammatory processes [34] .

In this study, we demonstrated that DMF treatment inhibited the nuclear translocation of NF-κB, representing a functional system to regulate neuroinflammation in response to migraine.

NF-κB activation is correlated with an upregulation of proinflammatory mediators such as tumor necrosis factor (TNF)-α, interleukins, and prostaglandins as well as nitrosative stress. In this study, DMF treatment significantly reduced iNOS and COX-2 expressions.

In response to inflammatory and oxidative stimuli, upregulation of Nrf-2 signaling inhibits the overproduction of proinflammatory cytokines and chemokines as well as limiting the activation of NF-κB [70]. In normal conditions, Nrf-2 is located in the cytosol, under oxidative stressors Nrf2 translocate to the nucleus activating antioxidative genes [71]. In this study, we observed that NTG administration significantly increased nuclear Nrf-2 expression in mice as well as the expression Nrf-2-regulated phase II enzymes, Mn-SOD and HO-1 [72]. DMF treatments markedly upregulated the antioxidant enzymes, demonstrating its important role in the reduction of oxidative stress related to migraine. Moreover, our study clearly showed that DFM in a pre-treatment condition was unable to prevent NTG tissue damage, validating its use only as a post-treatment during migraine attack.

## Conclusion

In this study, we demonstrated for the first time the DMF potential for treatment of migraine, modulating the inflammatory state and strengthening the antioxidant Nrf-2 transcriptional pathway, as confirmed by the in vitro study. Moreover, considering that anxiety plays an important fact in migraine risk, detecting anxiety symptoms and implementing pharmacological and not treatments targeting Nrf-2/NF-κB pathway could improve headache control and patient’s quality of life. In conclusion, DMF could be considered as promising therapeutic approach in counteract the inflammation and oxidative stress associated to migraine.

## Supplementary information

**Additional file 1: Figure S1.** Evaluation of possible effects of NTG-vehicle on Nrf-2 pathway expression. The expression of Nrf-2, Mn-SOD and HO-1was performed by Western Blot analysis in whole brain with the rostral cervical spinal cord samples of mice. Not significantly differences were observed in in Nrf-2 (A), Mn-SOD (B) and HO-1 (C) expression in sham+Sumatriptan and in sham+DMF groups compared to control. Respectively (A), F value = 2.913, (B) (F value = 3.001) and (C), F value = 0.377. Data are means ± SEM of 10 mice for each group. A representative blot of lysates obtained from each group is shown and densitometry analysis of all animals is reported (*n* = 10 mice from each group). One-Way ANOVA followed by Bonferroni post- test. Statistical significance was not observed.

**Additional file 2:** **Figure S2.** Evaluation of possible effects of NTG-vehicle on NF-κB pathway expression. The expression of NF-κB, IκB-α, iNOS and COX-2 was performed by Western Blot analysis in whole brain with the rostral cervical spinal cord samples of mice. Not differences were observed in the nuclear translocation of NF-κB (A) and in cytosolic IκB-α (B) degradation in sham+Sumatriptan and sham+DMF compared to control group. Respectively (A), F value = 6.49 and (B), F value = 1.75. iNOS expression was similar in all control groups (C), F value = 1.39, while Cox-2 expression augmented, but not significantly, in sham+Sumatriptan and sham+DMF compared to control (D), F value = 1.26. Statistical significance was not observed.

## Data Availability

The datasets used and/or analyzed during the current study are available from the corresponding author on reasonable request.

## Abbreviations

5-HT: Serotonin 5-hydroxytryptamine serotonin
COX-2: Cyclooxygenase 2
DETA/NO: Diethylenetriamine/nitric oxide
DMF: Dimethylfumarate
EPM: Elevated plus maze
FAE: Fumaric acid esters
GPCR: Calcitonin gene-related peptide
HO-1: Heme-oxygenase-1
INOS: Inducible nitrite oxide synthase
IκBα: Nuclear factor of kappa light polypeptide gene enhancer in B-cells inhibitor alpha
MMF: Monomethylfumarate
Mn-SOD: Manganese superoxide dismutase
MS: Multiple sclerosis
NF-кB: Nuclear factor kappa-light-chain-enhancer of activated B cells subunit p65
NO: Nitric oxide
Nrf-2: Nuclear factor erythroid-2-related factor-2
NSAID: Nonsteroidal anti-inflammatory drugs
NTG: Nitroglycerin
ROS: Reactive oxygen species
SpV: Spinal trigeminal nucleus
TGVS: Trigemino vascular system
