# Editorial expression of concern: Dimethyl fumarate alleviates the nitroglycerin (NTG)-induced migraine in mice

**DOI:** 10.1186/s12974-026-04087-5

**Published:** 2026-10-13

**Authors:** Giovanna Casili, Marika Lanza, Alessia Filippone, Michela Campolo, Irene Paterniti, Salvatore Cuzzocrea, Emanuela Esposito

**Affiliations:** 1 Department of Chemical, Biological, Pharmaceutical and Environmental Sciences, University of Messina, Viale Ferdinando Stagno D ‘Alcontres, 31, Messina, 98166 Italy; 2 Department of Pharmacological and Physiological Science, Saint Louis University, Room M 36-1402 South Grand Blvd, St. Louis, MO 63104 USA

**Journal of Neuroinflammation 17:59 (2020)**

**https://doi.org/10.1186/s12974-020-01736-1**

The Editors-in-Chief would like to alert the readers that a concern has been raised regarding some highly similar features between Fig. 8 panels D and E. Readers are therefore advised to interpret these results with caution.

Giovanna Casili has stated on behalf of all authors that they do not agree to this Editorial Expression of Concern.

