# Supplementary figures and images for: Dimethyl fumarate alleviates the nitroglycerin (NTG)-induced migraine in mice
Source: J Neuroinflammation. 2020 Feb 17;17:59. doi: 10.1186/s12974-020-01736-1 (PMC7469611; doi:10.1186/s12974-020-01736-1)

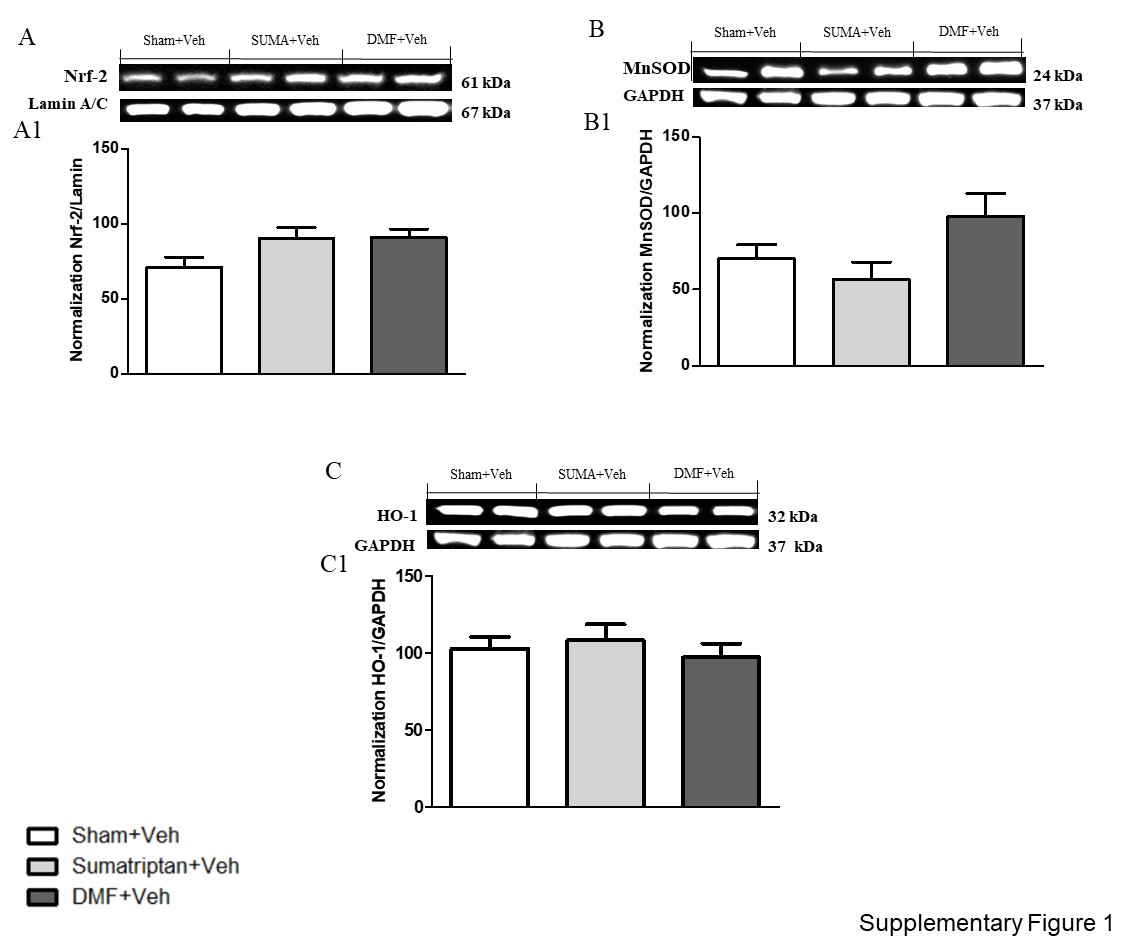

Supplement: Supplementary file 1 — Additional file 1: Figure S1. Evaluation of possible effects of NTG-vehicle on Nrf-2 pathway expression. The expression of Nrf-2, Mn-SOD and HO-1was performed by Western Blot analysis in whole brain with the rostral cervical spinal cord samples of mice. Not significantly differences were observed in in Nrf-2 (A), Mn-SOD (B) and HO-1 (C) expression in sham+Sumatriptan and in sham+DMF groups compared to control. Respectively (A), F value = 2.913, (B) (F value = 3.001) and (C), F value = 0.377. Data are means ± SEM of 10 mice for each group. A representative blot of lysates obtained from each group is shown and densitometry analysis of all animals is reported (n = 10 mice from each group). One-Way ANOVA followed by Bonferroni post- test. Statistical significance was not observed. [file 12974_2020_1736_MOESM1_ESM.jpg]

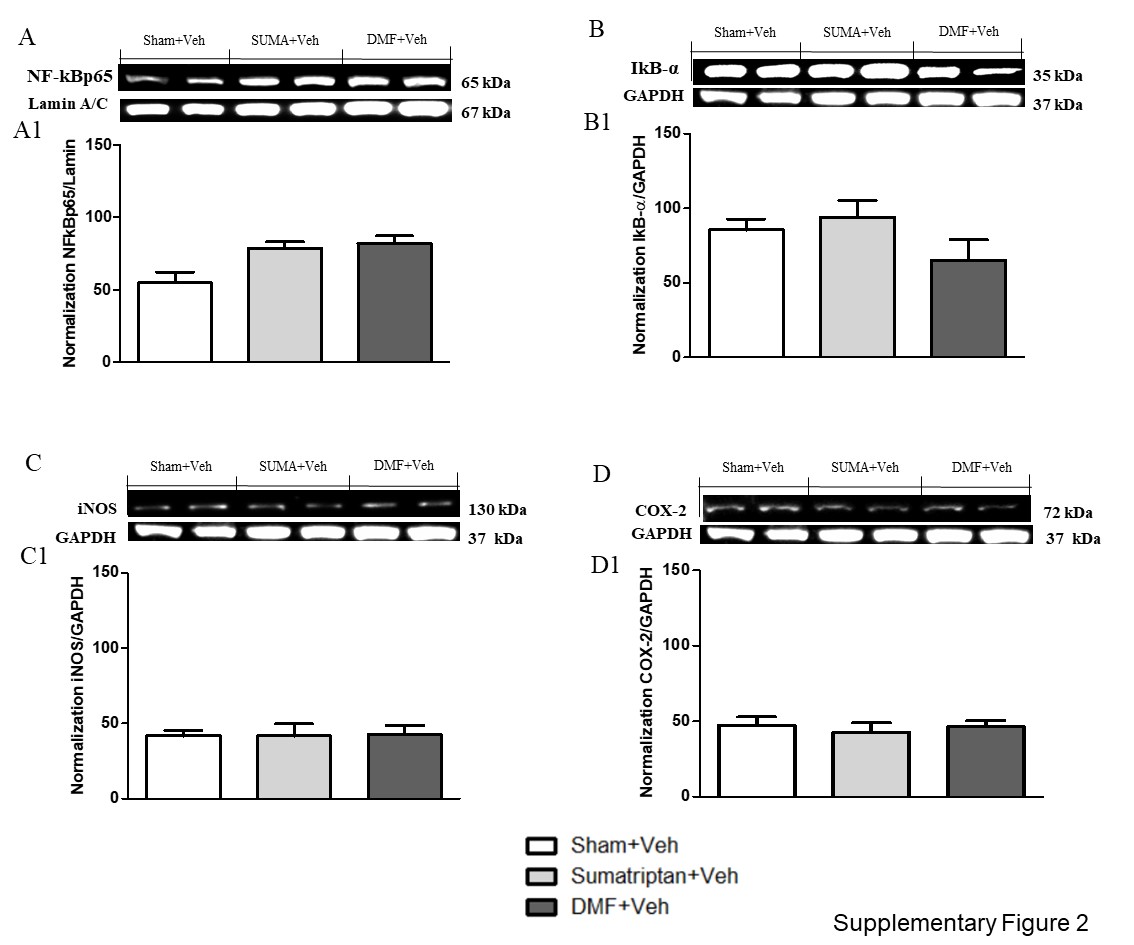

Supplement: Supplementary file 2 — Additional file 2: Figure S2. Evaluation of possible effects of NTG-vehicle on NF-κB pathway expression. The expression of NF-κB, IκB-α, iNOS and COX-2 was performed by Western Blot analysis in whole brain with the rostral cervical spinal cord samples of mice. Not differences were observed in the nuclear translocation of NF-κB (A) and in cytosolic IκB-α (B) degradation in sham+Sumatriptan and sham+DMF compared to control group. Respectively (A), F value = 6.49 and (B), F value = 1.75. iNOS expression was similar in all control groups (C), F value = 1.39, while Cox-2 expression augmented, but not significantly, in sham+Sumatriptan and sham+DMF compared to control (D), F value = 1.26. Statistical significance was not observed. [file 12974_2020_1736_MOESM2_ESM.jpg]
